# 

**Published:** 1912-02-15

**Authors:** 


### ANNOUNCEMENTS.

Chapin A. Harris Memorial Fund.—The commitee appointed by the Maryland State Dental Association in charge of the Chapin A. Harris Memorial Fund call your attention to the desire on the part of the dental profession to commemorate in a suitable manner their appreciation of the services rendered by this distinguished man in the effort to elevate the profession of dentistry.

While not a Marylander by birth, his life-work was accomplished in this state, which was his home, and it was in the city of Baltimore that he established the first dental school, thus making dentistry a profession.

Connecticut has recognized and honored the name of Horace H. Hayden, a co-worker with Harris, by erecting a handsome monument to his memory at Windsor.

We are now asking contributions from dental societies, colleges, and members of the profession at large, to aid in erecting a suitable monument to the memory of Chapin A. Harris, the Founder of Professional Dentistry.

Those wishing to contribute will kindly send to Dr. H. A. Wilson, treas., Calvert Bank Bldg., Baltimore, Md.

W. G. Foster, *Chairman*,
W. W. Dunbracco, *Sec'y*,
M. G. Sykes,
B. Holly Smith,
T. O. Heatwole,
J. W. Smith,
H. A. Wilson, *Treas.*

South Dakota State Dental Society. The thirtieth annual meeting of the South Dakota State Dental Society will be held at Sioux Falls, May 14 and 15, 1912. Adoption of a new constitution and by-laws will take place at this meeting.

J. D. Donahoe, *Sec'y,*
Sioux Falls, S. D.

---

Kansas State Dental Association. The forty-first annual convention of the Kansas State Dental Association will be held in the city hall at Salina, Kans., April 23, 24 and 25, 1912.

S. S. Noble, *President.*
Wichita, Kans.

